# Advancing Genomics for Drug Development and Safety Evaluation

**DOI:** 10.1155/2018/3126820

**Published:** 2018-05-23

**Authors:** Zhichao Liu, Joshua Xu, Zhining Wen

**Affiliations:** ^1^Division of Bioinformatics and Biostatistics, National Center for Toxicological Research, U.S. Food and Drug Administration, Jeffersonville, IN, USA; ^2^Sichuan University, Chengdu, China

This special issue is focused on advancing genomics for drug development and safety evaluation. Gaining wide adoption in biomedical fields, genomic technologies have tremendously improved our molecular understanding of disease etiology and pathogenesis. Furthermore, the accumulated genomic datasets allow us to articulate new hypothesis, revisit and rethink the conventional paradigm for drug development and safety evaluation, and generate more efficient tools to promote biomedical researches.

Cancer genomics has made a great progress to uncover the underlying mechanism of tumorigenesis. Consequently, a lot of cancer genomic biomarkers have been developed for improving cancer diagnosis and prognosis. Questions arise on how the biomarkers developed from one study could be extrapolated to another. The advancement of machine learning technologies provides us a great opportunity to address these crucial questions such as model transfer and data integrity issues in the cancer field. R. Jing et al. proposed an ensemble method with voting protocols to integrate multiple machine learning algorithms to predict cancer outcome. It was indicated the proposed ensemble approaches could greatly improve the robustness and stability of cancer prediction modeling.

Genomic technologies such as next-generation sequencing provided an unprecedented resolution to better understand complex regulatory relationship among different genetic elements. The consortium efforts such as The Cancer Genomics Atlas (TCGA) not only covers diverse cancer subtypes but also includes a lot of genomic elements and events (i.e., miRNA, copy number variation, and DNA methylation). Accordingly, the approaches to integrate these genomic elements to decipher the complex regulatory relationship tailored to different cancer mechanisms are urgently needed. J. Xue et al. applied graphical lasso models (GLMs) to predict miRNA-mRNA relationship for three cancer types including acute myeloid leukemia (AML), breast invasive carcinoma (BRCA), and kidney renal clear cell carcinoma (KIRC) in TCGA. The results suggested that the proposed network approaches could improve the prediction performance to enrich more tumorigenesis-related miRNA-mRNA relationship.

Shift work is a common social issue due to the rapid pace of modern lifestyle. The disruption in circadian clock system leads to a lot of health concerns and increases the risk to develop serious diseases including sleep disorders, metabolic disorders, psychiatric disorders, and even cancers. S. Khan et al. summarized the shift work-related disorders and elaborated on the potential risk factors and mechanisms with a comprehensive literature survey. It is very interesting that some gene expressions and genetic variants were identified for playing an important role in shift work-related health disorders, which paves a way to further uncover the genetic contribution to shift work-related diseases and develops therapy to control and relieve the syndromes.

Protein classification based on organisms is a hot topic in microbiology. Considering huge amount of protein sequencing data that were generated in the past two decades, supplicated model development strategies and novel approaches are needed to categorize the proteins from different organisms. H.-B. Guo et al. developed novel fingerprints based on protein distribution densities in the LD space and implemented a machine learning framework to improve the accuracy of protein organism classification. The proposed approach could be potentially applied to microbiome field and related disciplines.

A lot of drug candidates in the clinical trial failed due to unexpected adverse drug reactions (ADRs). ADR especially idiosyncratic adverse drug reaction (IADR) is difficult to study. Drug-induced myopathy as an IADR is unpredictable and dose independent. To better understand the causes for drug-induced myopathy, D. Li et al. developed a systematic approach to integrating different data profiles including chemical structure information, drug-protein relationship, side effects, and transcriptomic data profiles to identify the risk factors for drug-induced myopathy. This study sets a great example for fusing the genomic data with other types of data profiles to elucidate the hidden genotype-phenotype relationship. Furthermore, the key factors including structure alerts could be applied to develop predictive models for early detection and prevention of drug-induced myopathy.

Many aspects of genomics were not covered in this special issue. For example, the reproducibility of genomic studies, inconsistent results from different data analysis pipelines, and data storage are also of great importance for better utilization of genomic technology to promote and improve public health. We hope this special issue could serve as a trigger to stimulate the common interest in the community for advancing genomic technologies.

*Zhichao Liu* *Joshua Xu* *Zhining Wen*

